# Matrix-assisted laser desorption/ionization mass spectrometry imaging analysis revealed the spatial distribution of metabolites during *Ziziphi Spinosae Semen* at different growth periods

**DOI:** 10.3389/fpls.2025.1510310

**Published:** 2025-02-21

**Authors:** Mengwei Zhao, Zhao Liu, Jiemin Wang, Yuting Liu, Yuping Yan, Ning Liu, Jianming Jiang, Yongxing Song, Huigai Sun, Donglai Ma

**Affiliations:** ^1^ College of Pharmacy, Hebei University of Chinese Medicine, Shijiazhuang, China; ^2^ Traditional Chinese Medicine Processing Technology Innovation Center of Hebei Province, Shijiazhuang, China; ^3^ The First Affiliated Hospital of Hebei University of Traditional Chinese Medicine, Shijiazhuang, China; ^4^ International Joint Research Center on Resource Utilization and Quality Evaluation of Traditional Chinese Medicine of Hebei Province, Shijiazhuang, China

**Keywords:** *Ziziphi Spinosae Semen*, MALDI, growth in different periods, potential quality markers, PCA

## Abstract

*Ziziphi Spinosae Semen* (ZSS), a medicinal food, is one of the most popular Chinese medicinal herbs, known for its rich bioactive ingredients, such as terpenoids and flavonoids. The content of these bioactive ingredients in ZSS varies with age. However, the distribution of these bioactive ingredients throughout the developmental phases of ZSS—white maturity (S1), firm ripening (S2), and full ripening (S3)—and within specific tissues like the cotyledon, endosperm, and radicle, has not yet been determined. This research aimed to analyze the spatial distribution of key quality markers in ZSS and elucidate the metabolite quality characteristics throughout its growth stages. Utilizing matrix-assisted laser desorption/ionization-mass spectrometry imaging (MALDI-MSI), 25 bioactive ingredients were identified and visualized in ZSS, including three terpenoids, seven flavonoids, four alkaloids, and eleven amino acids. The MALDI images revealed distinct spatial variations in the distribution of these bioactive ingredients across different regions of ZSS tissues and different growth stages. Moreover, as ZSS matured, the distribution area of specific bioactive ingredients significantly increased, encompassing three terpenoids, six flavonoids, two alkaloids, and seven amino acids. Utilizing ion imagery, Principal Component Analysis (PCA) coupled with Orthogonal Partial Least Squares Discriminant Analysis (OPLS-DA) effectively differentiated samples from various developmental periods. Additionally, nine major bioactive ingredients were identified as potential quality markers for ZSS. This research demonstrates that the content and distribution of bioactive components in ZSS change during growth. The analysis provided by MALDI-MSI offers an experimental basis for understanding the accumulation and distribution of bioactive components during the maturation of ZSS.

## 1 Introduction

*Ziziphus jujube* Mill. var. sp*inosa* (Bunge) Hu ex H. F. Chou is a deciduous shrub or small tree whose fruit is edible (Yang et al., 2023). The seed of this plant, known as Ziziphi Spinosae Semen (ZSS), holds significant edible and medicinal value and is highly regarded in the food and pharmaceutical industries (Wang et al., 2023a, b). ZSS is commonly used to prepare tea and porridge and is often exported as raw material (Yan et al., 2020). Its medicinal properties were first documented in the Shennong Herbal Scripture and have been widely recognized across East Asia (Wu et al., 2023). ZSS is known for its sedative and calming effects. Modern pharmacological studies have confirmed its strong anxiolytic, antidepressant, and neuro-protective effects (Wang et al., 2022; Bian et al., 2021). ZSS contains various bioactive substances, including terpenoids, flavonoids, alkaloids, and amino acids (Shi et al., 2024; Pan et al., 2023). However, the content of these bio-active ingredients varies at different developmental stages (Xue et al., 2022), and understanding the spatial distribution changes of these important bioactive ingredients during ZSS development is crucial for researching their physiological roles, nutritional values, and potential functional benefits.

To investigate the distribution of bioactive ingredients in ZSS, a method that reveals their spatial distribution must be developed (Li et al., 2022a). Mass spectrometry imaging (MSI) provides chemical distribution information by Detecting the spatial distribution of compounds within the ZSS seed body (Huang et al., 2022). With optical microscopy, MSI can link morphological features to chemical analysis, offering non-target, label-free, and multiplexing methods for molecular imaging (Chen et al., 2023). Recently, MSI has become an essential tool for capturing the spatial accumulation and localization of plant bioactive ingredients (Jiang et al., 2022a). For example, Li et al. identified the spatial distribution of components such as paeoniflorin and albiflorin in the roots of *Paeonia suffruticosa* and *Paeonia lactiflora* using the MALDI-MSI technique (Li et al., 2021). Zhao et al. used MALDI-MSI to identify the spatial distribution of endogenous molecules in wolfberry fruit at different developmental stages (Zhao et al., 2021). Researchers have also characterized the spatial distribution of components in *Coptis chinensis* rhizome (He et al., 2022) and *Dendrobium nobile* (Liu et al., 2023a), providing references for quality control and separation.

In this research, MALDI-MSI was employed to perform a spatial analysis of bioactive ingredients in ZSS samples collected at various developmental stages. We focused on terpenoids, flavonoids, alkaloids, and amino acids. Subsequently, multivariable statistical methods were applied to identify the key bioactive ingredients exhibiting significant variation across different periods of ZSS development. The mass spectrometry imaging results provide a better understanding of the chemical changes in ZSS during growth.

## 2 Materials and methods

### 2.1 Chemicals and plant materials

LC-MS grade methanol, ethanol, acetonitrile, formic acid, 0.1% (w/v) poly(L-lysine) hydrobromide solution, and gelatin were obtained from Sigma-Aldrich (Shanghai, China). Analytical grade ammonium acetate, chloral hydrate, phloroglucinol, iodine, sulfuric acid, and ethanol were obtained from Sinopharm Chemical Reagent Co., Ltd. (Beijing, China). The optimum cutting temperature compound was obtained from Leica (Nussloch, Germany). Water was purified with a Milli-Q filtration system (Millipore, Bedford, MA, USA). ZSS at different developmental stages—white maturity (S1), firm ripening (S2), and full ripening (S3)-was provided by Wennuanhe, Xingtai City, Hebei Province, China.

### 2.2 Preparation of low molecular weight standard compounds

Standard solutions of various terpenoids, flavonoids, alkaloids, fatty acids, amino acids, and other low molecular weight compounds, including jujuboside A, jujuboside B, sanjoinenine, spinosin, 6’’’-feruloylspinosin, apigenin-6-glucosyl-7-O-methyl ether (AGOME), vicenin-2, 6’’-p-coumaroylspinosin, 6’’’-sinapoylspinosin, (2S, 3S)-N-[(1S)-1-carbamoyl-3-menthyl-butyl]-2-[[(2S)-2-dimethylamino-3-phenyl-propanoyl]amino]-3-(4-formylphenoxy)-4-methyl-valeramide (CMBDPM), DL-nuciferine, amphibine D, ortho-nornuciferine, glutamic acid, proline, lysine, phenylalanine, methionine, L-isoleucine, arginine, serine, (S)-coclaurine, eleutheroside A, adenosine, and catechin, were prepared at a concentration of 1 mmol in deionized water for MALDI-TOF MSI detection. To prepare the mixed standard solution, 25 low molecular weight compounds were dissolved in deionized water at a concentration of 10 mM and diluted to the desired concentrations. One microliter of each standard solution was spotted on an AnchorChip target plate for MALDI-TOF MS detection.

### 2.3 Tissue sectioning

ZSS tissues at different developmental stages were cryo-sectioned at −20°C into 20-μm thick slices using a Leica CM1860 cryostat (Leica Microsystems, Wetzlar, Germany). The serial tissue slices were then immediately thaw-mounted on the conductive sides of indium tin oxide-coated microscope glass slides (Bruker Daltonics, Billerica, MA, USA) (batch number 20220727A, specific resistance 6 Ω).

### 2.4 Matrix coating

MALDI matrices were applied using an ImagePrep electronic matrix sprayer (Bruker Daltonics, Bremen, Germany). The detection matrix was 12 mg/mL 2-MBT in methanol: water (4:1, v/v) containing 0.2% trifluoroacetic acid. The matrix spraying was conducted according to the instrument manufacturer’s method with some modifications (Ma et al., 2024; Du et al., 2020). During the matrix spraying, the incubation time was 30 s, the wetness was 40%, and other parameters were set to default. Sprayed tissue sections were prepared for MALDI-MSI analysis, and an Epson Perfection V550 Photo Scanner (Epson (China) Co., Ltd., Beijing, China) was used to capture optical images of the tissue sections.

### 2.5 MALDI-MS

The detection matrix was 12 mg/mL 2-MBT in methanol: water (4:1, v/v) containing 0.2% trifluoroacetic acid. The matrix spraying was conducted according to the instrument manufacturer’s method with some modifications (Ma et al., 2024; Du et al., 2020). During the matrix spraying, the incubation time was 30 s, the wetness was 40%, and other parameters were set to default. Sprayed tissue sections were prepared for all profiling and imaging experiments were performed using an Autoflex Speed MALDI-TOF/TOF-MS (Bruker Daltonics, Billerica, MA, USA). The MALDI source was equipped with a 2,000 Hz solid-state Smartbeam Nd: YAG UV laser (355 nm, Azura Laser AG, Bremen, Germany). Mass spectra were acquired over a mass range of *m/z* 100 to 1,500 in positive-ion mode with broadband detection, obtained from 40 scans at 500 laser shots each. For imaging data acquisition, 100 μm laser raster step sizes were used to detect endogenous low molecular weight compounds in the ZSS tissue sections, with each scan (pixel) accumulated from 500 laser shots.

Mass calibration employed a standard mixture for external calibration, comprising peptides with defined m/z values: bradykinin 1–7 ([M+H]^+^, *m/z*=757.40), angiotensin II ([M+H]^+^, *m/z*=1046.54), angiotensin I ([M+H]^+^, *m/z*=1296.69), substance P ([M+H]^+^, *m/z*=1347.74), and bombesin ([M+H]^+^, *m/z*=1619.82). Internal calibration referenced the matrix ion 2-MBT ([M+H]^+^, *m/z*=167.99) and a peptide standard ([M+H]^+^, *m/z*=1349.69), with the intensity of the peptide standard normalizing the spectral data. Calibration was conducted in cubic-enhanced mode to ensure the accuracy of mass measurements. The cubic-enhanced mode was chosen for both external and internal mass calibration processing. The data were based on three biological replicates, and each biological replicate corresponded to three technical replicates.

Using Bruker’s FlexImaging 4.1 software (https://www.bruker.com/zh/services/software-downloads.html), a correction pen was used to mark the “teaching points” (typically three points) around a tissue section for correct UV laser positioning for spectral acquisition. The MALDI-TOF mass spectra were processed with total ion current normalization, and the signal intensity of each imaging data point was represented as normalized intensity. MALDI-MSI analysis, and an Epson Perfection V550 Photo Scanner (Epson (China) Co., Ltd., Beijing, China) was used to capture optical images of the tissue sections.

### 2.6 Data analysis

After data collection using MALDI-TOF-MSI, the spectral files were processed using FlexAnalysis 3.4 software (Bruker Daltonics GmbH, Bremen, Germany) on cryo-sectioned tissue. In each sample’s measured area, regions such as cotyledon, radicle, and endosperm were defined according to the botanical structure of ZSS. Regions of interest containing features like the cotyledon, radicle, and endosperm were identified. Average spectra from these regions were used to set peak integration ranges. External and internal calibrations were conducted during acquisition to correct for instrumental issues. Post-export, m/z values were aligned across replicates to match compounds across samples. The aligned data were integrated into a CSV file. SPSS26.0 software (https://www.ibm.com/cn-zh/spss) was used for data analysis, and the statistical results were expressed as x̄ ± s. In order to evaluate the significant differences between groups, Duncan’s new multiple range test (DNMRT) was used to compare the mean values of different groups on the basis of the data conforming to normal distribution and homogeneity of variance. First, arrange all the averages in descending order. The maximum average is then marked as a and compared with the adjacent average. If the difference is not significant, the adjacent mean is also marked as a; If significant, the next mean is labeled b. This process is repeated until all averages are marked. Finally, the mean of the same markers indicated no significant difference, while the mean of different markers indicated significant difference, and the significance level was set at P < 0.05. Use GraphPadPrism9 software (https://www.graphpad.com/scientific-software/prism/www.graphpad.com/scientific-software/prism/) to draw the column chart. Prior to multivariate analysis, the data were normalized using mean-centering and unit variance scaling. PCA and OPLS-DA were then applied using SIMCA 14.1 (https://www.sartorius.com/en/products/process-analytical-technology/data-analytics-software/mvda-software/simca) to differentiate samples with various quality parameters and identify potential quality-associated markers to differentiate samples and identify quality markers (Hou et al., 2022).

## 3 Results

### 3.1 Morphological characteristics of ZSS

**Figure 1:**
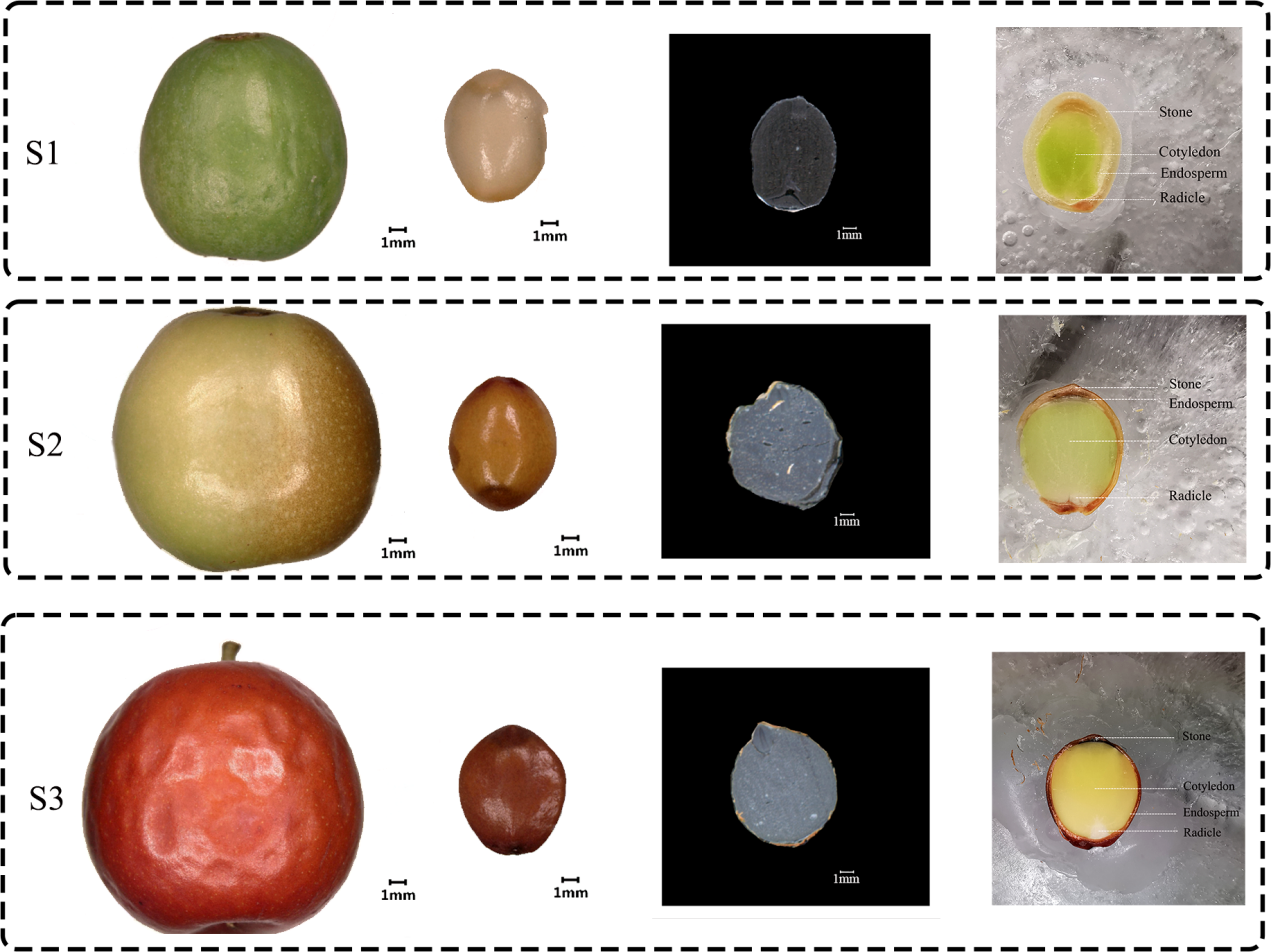
Macroscopic features of surface and transverse sections at the fruit and seed of Ziziphus jujube Mill. var. spinosa growth in different periods. S1 was the White maturity stage, S2 was the firm ripening stage and S3 was the full ripening stage.

The morphological analysis of Ziziphus jujube Mill. var. spinosa fruit at different developmental stages revealed distinct characteristics (**Figure 1**). At the white maturity (S1) stage, the fruit surface was green, with smaller flesh and a hard texture. At the firm ripening (S2) stage, the fruit was larger, mostly green with some red, and had a firmer texture. At the full ripening (S3) stage, the fruit surface was completely red, and the flesh was soft and fragrant. The extrinsic feature of the ZSS seed is oblate and oval with a raised vertical line in the middle. The seed surface at S1 is green and comparatively shriveled, at S2 is yellow to yellow-brown, and at S3 is relatively plump and shiny with a purplish-red or purplish-brown hue. To further understand the internal structure of ZSS, this research freeze-dried and sliced ZSS in different periods (**Figure 1**). The results of sectioning showed that the tissues of ZSS were endosperm, radicle, and cotyledon from outside to inside.

### 3.2 *In situ* metabolite profiling of ZSS at different growth stages using MALDI-MSI

The integrated metabolite profiles of ZSS at different growth stages were generated using MALDI-MSI (**Supplementary Figure 1**). Positive ions were mainly detected in the *m/z* 100-1200 range. By screening ions based on ZSS standards, literature reviews, and other databases, 25 components, including flavones, saponins, alkaloids, and amino acids, were selected for MALDI-MSI analysis. These compounds, representative of four different classes of bioactive components (terpenoids, flavonoids, alkaloids, and amino acids), are detailed in **Table 1**.

**Table 1:** Identification of differential metabolites of ZSS at different growth stages tissue sections using MALDI-MSI.

NO.	Metabolite	Formula	Ion formula	Theoretical *m/z*	Observed *m/z*	ppm	Peak area			Fragment ions m/z
							S1	S2	S3	
1	Jujuboside A	C_58_H_94_O_26_	[M+K]^+^	1245.5665	1245.566	0.399	9557.76	11692.78	15613.67	927, 603, 473, 279, 67
2	Jujuboside B	C_52_H_84_O_21_	[M+H]^+^	1045.5578	1045.558	0.191	2386.66	1590.22	13426.11	942, 763, 455, 305, 119, 39
3	Sanjoinenine	C_29_H_35_N_3_O_4_	[M+H]^+^	490.2700	490.270	0	2607.82	539.22	604.78	242, 131
4	Spinosin	C_28_H_32_O_15_	[M+K]^+^	647.1373	647.137	0.463	2768.21	7100.44	11754.22	351, 297
5	6’’’-Feruloylspinosin	C_38_H_40_O_18_	[M+H]^+^	785.2287	785.228	0.891	13060.62	27538.33	48018.56	327, 351, 177, 297, 429, 665, 785
6	AGOME	C_22_H_22_O_10_	[M+Na]^+^	469.1105	469.111	1.065	2722.81	3132.78	3870.56	—
7	Vicenin-2	C_27_H_30_O_15_	[M+Na]^+^	617.1477	617.147	1.134	61659.21	56699.44	83914.44	577, 385, 309, 163, 89
8	6’’-p-Coumaroylspinosin	C_38_H_40_O_17_	[M+Na]^+^	791.2158	791.216	0.253	1408.02	1317.33	1794.67	447, 429, 351,
9	6’’’-Sinapoylspinosin	C_39_H_42_O_19_	[M+H]^+^	799.2440	799.244	0	9387.62	6444.89	15079.78	635, 447, 429, 393, 351, 327, 297, 207
10	CMBDPM	C_30_H_42_N_4_O_5_	[M+H]^+^	539.3228	539.323	0.371	12439.43	13181.00	10382.33	——
11	Dl-Nuciferine	C_19_H_21_NO_2_	[M+H]^+^	296.1645	296.164	1.688	1008.33	1166.78	1192.22	265, 296, 250; 234; 237
12	Amphibine D	C_36_H_49_N_5_O_5_	[M+H]^+^	632.3806	632.381	0.632	5116.94	1855.89	2568.00	148, 289, 261,316, 344, 632
13	Ortho-nornuciferine	C_18_H_19_NO_2_	[M+Na]^+^	304.1308	304.131	0.675	3384.81	3081.56	2141.22	——
14	Glutamic acid	C_5_H_9_NO_4_	[M+K]^+^	186.0163	186.016	1.613	2048.01	9352.22	17058.33	85, 56, 42
15	Proline	C_5_H_9_NO_2_	[M+Na]^+^	138.0525	138.052	3.622	6193.61	3388.11	3366.89	70, 68
16	Lysine	C_6_H_14_N_2_O_2_	[M+K]^+^	185.0687	185.069	1.621	7797.41	30949.11	47886.33	84, 56
17	Phenylalanine	C_9_H_11_NO_2_	[M+H]^+^	166.0863	166.086	1.806	2044.50	5989.11	13959.22	166, 120, 103, 91
18	Methionine	C_5_H_11_O_2_NS	[M+Na]^+^	172.0403	172.040	1.744	3289.00	2520.89	2255.44	104, 56
19	L-Isoleucine	C_6_H_13_NO_2_	[M+K]^+^	170.0578	170.058	1.176	169468.31	163355.22	126331.78	70, 57, 41
20	Arginine	C_6_H_14_N_4_O_2_	[M+H]^+^	175.1190	175.119	0	60170.91	2072.33	772.44	70, 43
21	Serine	C_3_H_7_NO_3_	[M+H]^+^	106.0499	106.050	0.943	1722.01	1412.78	1149.78	88, 46
22	(S)-coclaurine	C_17_H_19_NO_3_	[M+K]^+^	324.0997	324.099	2.159	3764.01	1543.22	1301.11	257, 121, 79
23	Eleutheroside A	C_35_H_60_O_6_	[M+H]^+^	577.4463	577.446	0.519	1826.45	2161.33	2118.11	——
24	Adenosine	C_10_H_13_N_5_O_4_	[M+K]^+^	306.0599	306.060	0.327	3361.91	856.11	994.11	136, 119, 94, 92, 57
25	Catechin	C_15_H_14_O_6_	[M+H]^+^	291.0863	291.086	1.031	3962.30	2256.44	3375.78	259, 123, 51

### 3.3 Spatial distribution of terpenoids in ZSS at different growth stages using MALDI-MSI

**Figure 2:**
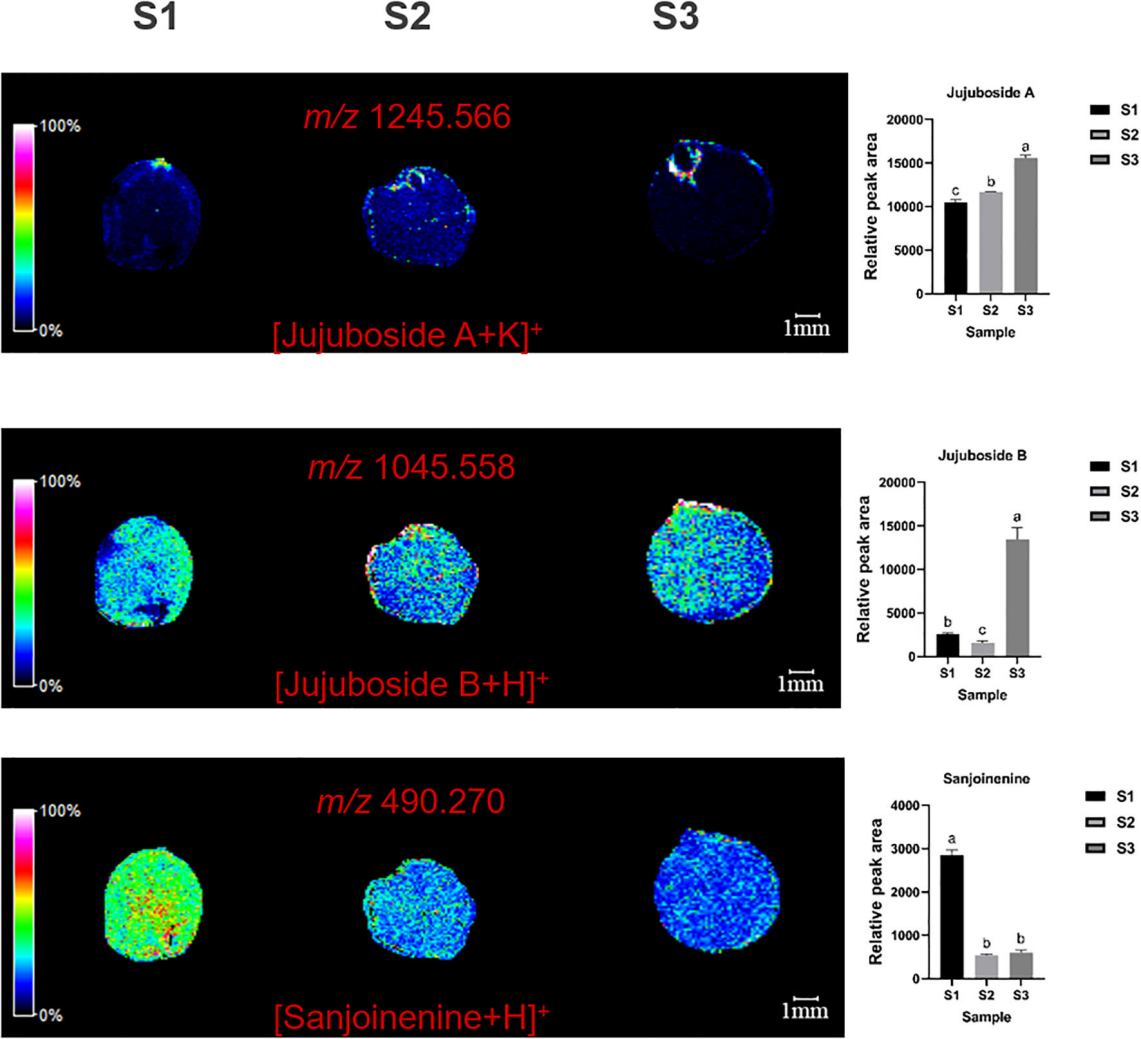
Selected ion maps of three terpenoids detected from the tissues of ZSS growth in different periods sections by MALDI-TOFTOF MS in the positive-ion mode using 2-MBT as the matrix. A color scale from white to blue indicates a high-to-low sign. S1 was the period of white maturity, S2 was the period of firm ripening and S3 was the period of full ripening.

MALDI imaging revealed the spatial distribution of terpenoids such as jujuboside A (*m/z*=1245.566, [M+K]^+^), jujuboside B (*m/z*=1045.558, [M+H]^+^), and sanjoinenine (*m/z*=490.270, [M+H]^+^) in ZSS. Jujuboside A was mainly concentrated in the radicle, while jujuboside B and sanjoinenine were primarily found in the cotyledons and endosperm (**Figure 2**).

The results indicated that jujuboside A content increased by 63.36% in S3 compared to S1, and by 22.34% in S2 compared to S1. This suggests that jujuboside A accumulation increases with plant growth. Jujuboside B content was 462.55% higher in S3 and 76.81% lower in S2 than in S1. Sanjoinenine content decreased by 33.37% in S2 compared to S1, but increased by 12.16% in S3 compared to S2 (**Table 1**). These results indicate that jujuboside B and sanjoinenine accumulation first decreases and then increases with growth.

### 3.4 Spatial distribution of flavonoids in ZSS at different growth stages using MALDI-MSI

**Figure 3:**
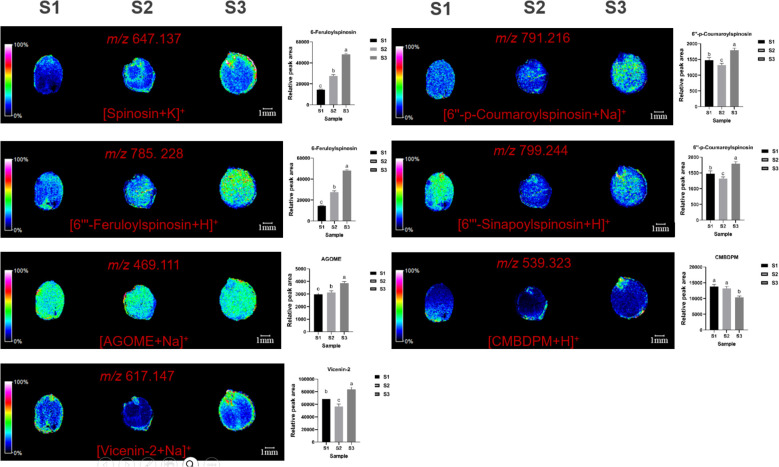
Selected ion maps of seven flavonoids detected from the tissues of ZSS growth in different periods sections by MALDI-TOFTOF MS in the positive-ion mode using 2-MBT as the matrix. A color scale from white to blue indicates a high-to-low sign. S1 was the period of white maturity, S2 was the period of firm ripening and S3 was the period of full ripening.

MALDI imaging also mapped the spatial distribution of flavonoids such as spinosin (*m/z*=647.137, [M+K]^+^), 6’’’-feruloylspinosin (*m/z*=785.228, [M+H]^+^), AGOME (*m/z*=469.111, [M+Na]^+^), vicenin-2 (*m/z*=617.147, [M+Na]^+^), 6’’-p-coumaroylspinosin (*m/z*=791.216, [M+Na]^+^), 6’’’-sinapoylspinosin (*m/z*=799.244, [M+H]^+^), and CMBDPM (*m/z*=539.323, [M+H]^+^). Spinosin was mainly concentrated in the radicle and endosperm, with content gradually increasing. 6’’’-feruloylspinosin was mainly found in the cotyledons. AGOME was distributed in the cotyledons and endosperm. Vicenin-2 was located primarily in the radicle and endosperm, while 6’’-p-coumaroylspinosin and 6’’’-sinapoylspinosin were mainly in the endosperm, and CMBDPM was mainly in the radicle (**Figure 3**).

Comparing S3 to S1, spinosin increased by 324.61%, 6’’’-feruloylspinosin by 267.66%, and AGOME by 42.15%. In S2, spinosin increased by 156.50%, 6’’’-feruloylspinosin by 110.85%, and AGOME by 15.06% compared to S1. This indicates that spinosin, 6’’’-feruloylspinosin, and AGOME gradually accumulate over time during plant growth. S3 also showed a 36.09% increase in vicenin-2, a 27.46% increase in 6’’-p-coumaroylspinosin, and a 60.63% increase in 6’’’-sinapoylspinosin compared to S1. In S2, vicenin-2 was 8.04% lower, 6’’-p-coumaroylspinosin was 6.44% lower, and 6’’’-sinapoylspinosin was 60.63% lower compared to S1. These results suggest that the accumulation of vicenin-2, 6’’-p-coumaroylspinosin, and 6’’’-sinapoylspinosin first increases over time. CMBDPM content in S3 was 16.54% lower than in S1, while in S2, it increased by 5.96%, indicating that CMBDPM increases during growth and development.

### 3.5 Detection of the spatial distribution of alkaloids in ZSS at different growth periods using MALDI-MSI

**Figure 4:**
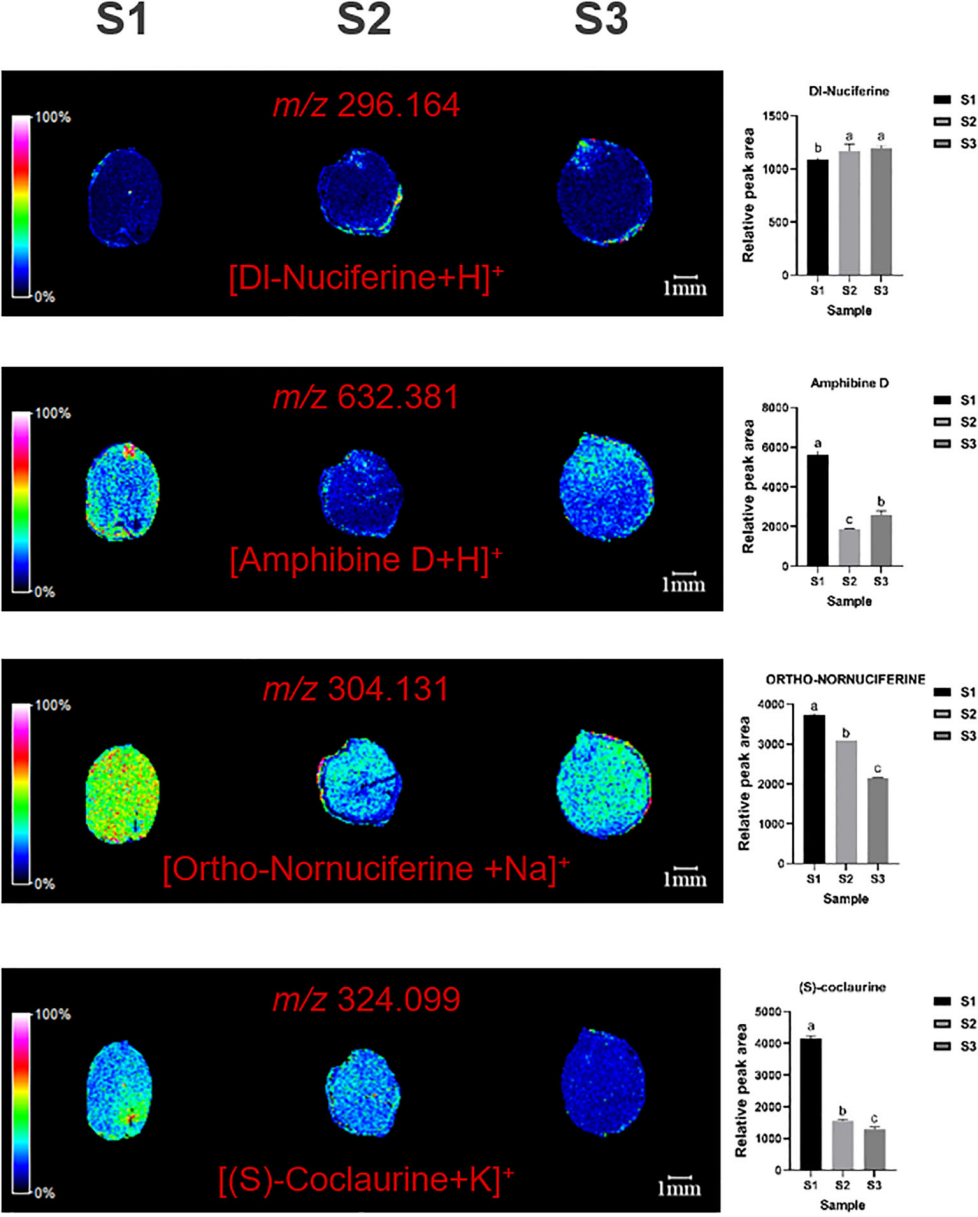
Selected ion maps of three alkaloids detected from the tissues of ZSS growth in different periods sections by MALDI-TOFTOF MS in the positive-ion mode using 2-MBT as the matrix. A color scale from white to blue indicates a high-to-low sign. S1 was the period of white maturity, S2 was the period of firm ripening and S3 was the period of full ripening.

MALDI imaging of ZSS identified the spatial distribution of several alkaloids, including dl-nuciferine (*m/z*=296.164, [M+H]^+^), amphibine D (*m/z*=632.381, [M+H]^+^), ortho-nornuciferine (*m/z*=304.131, [M+Na]^+^), and (S)-coclaurine (*m/z*=324.099, [M+K]^+^). dl-nuciferine was primarily concentrated in the radicle, amphibine D in the endosperm, and ortho-nornuciferine in both the endosperm and cotyledon. Initially, ortho-nornuciferine was widely distributed in the endosperm and cotyledons at S1. However, its content gradually increased in the endosperm and decreased in the cotyledons at S2 and S3. (S)-coclaurine is mainly distributed in the cotyledons, and gradually decreases over time, which was very low when present at S3 (**Figure 4**).

Specifically, S3 showed an 18.24% increase in dl-nuciferine, a 49.81% decrease in amphibine D, and a 36.74% decrease in ortho-nornuciferine compared to S1. In S2, dl-nuciferine increased by 15.71%, amphibine D by 63.73%, and ortho-nornuciferine by 8.96% compared to S1 (**Table 1**). These results indicate that dl-nuciferine content increases in the organism, with the fastest growth occurring between S1 and S2. Amphibine D content decreases initially and then increases, while Ortho-nornuciferine content gradually decreases, with the fastest decrease occurring between S2 and S3. S3 showed 65.43% reduce in (S)-coclaurine compared to S1. In S2, (S) -coclaurine was reduced by 59.00% compared to S1.

### 3.6 Detection of the spatial distribution of amino acids in ZSS at different growth periods using MALDI-MSI

**Figure 5:**
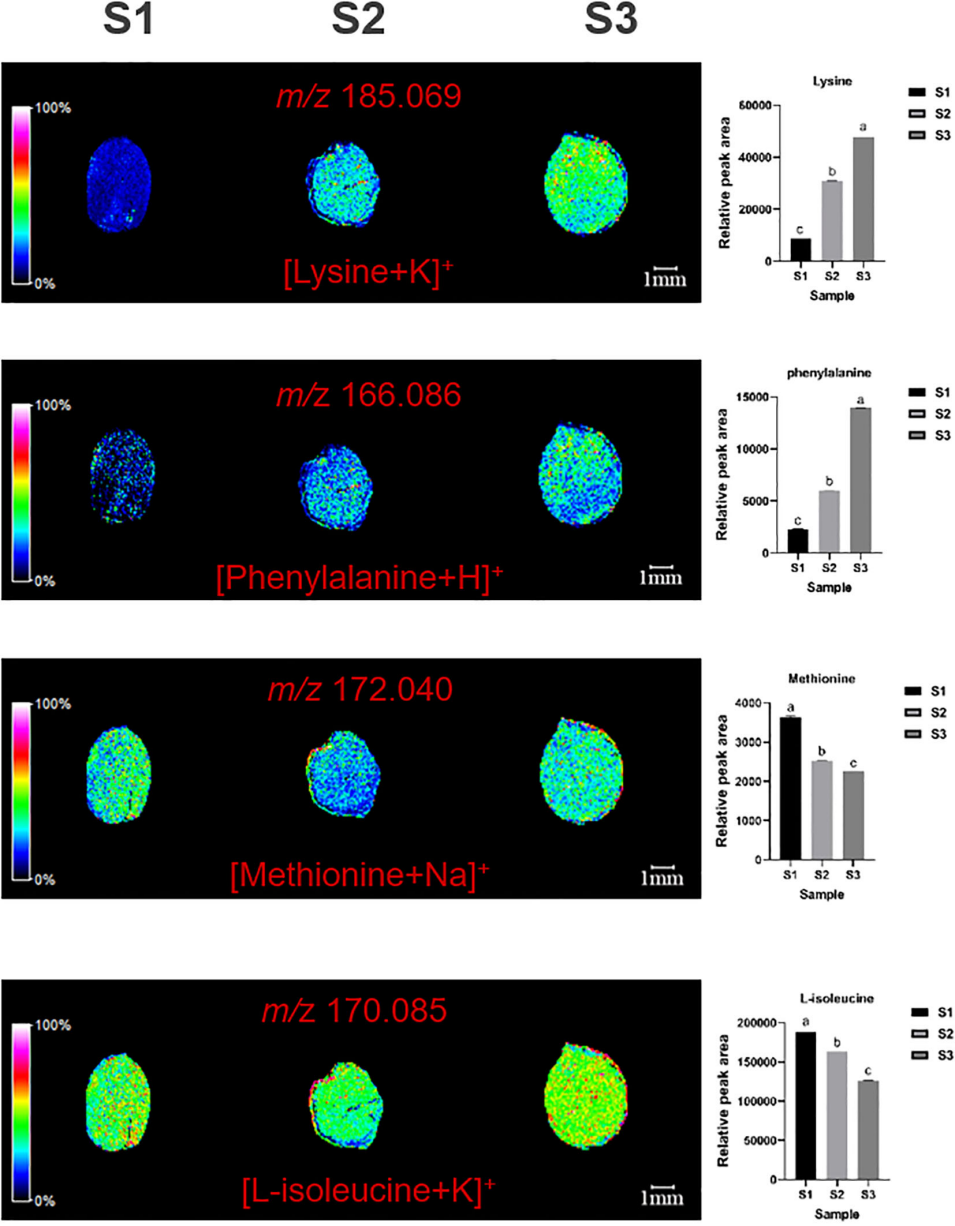
Selected ion maps of four essential amino-acids detected from the tissues of ZSS growth in different periods sections by MALDI-TOFTOF MS in the positive-ion mode using 2-MBT as the matrix. A color scale from white to blue indicates a high-to-low sign. S1 was the period of white maturity, S2 was the period of firm ripening and S3 was the period of full ripening.

MALDI imaging also revealed the spatial distribution of four essential amino-acids: lysine (*m/z*=185.069, [M+K]^+^), phenylalanine (*m/z*=166.086, [M+H]^+^), methionine (*m/z*=172.040, [M+Na]^+^), and L-isoleucine (*m/z*=170.058, [M+K]^+^) (**Figure 5**). Methionine was mainly distributed in cotyledons and radicles, with content first decreasing and then increasing. Lysine and phenylalanine were Mainly distributed in the cotyledons, with content gradually increasing. L-isoleucine is distributed throughout the ZSS, especially high in the endosperm (**Figure 5**).

Compared to S1, S3 showed reductions of 31.42% in methionine, and 25.45% in L-isoleucine. S2 showed reductions of 23.35% in methionine, and 3.61% in L-isoleucine, compared to S1. These findings indicate that the content of methionine and L-isoleucine generally decreases over time. Conversely, compared to S1, S3 showed increases of 514.13% in lysine, and 582.77% in phenylalanine. S2 showed increases of 296.92% in lysine, and 192.94% in phenylalanine compared to S1. This indicates that both of these amino acids increase during plant growth (**Table 1**).

**Figure 6:**
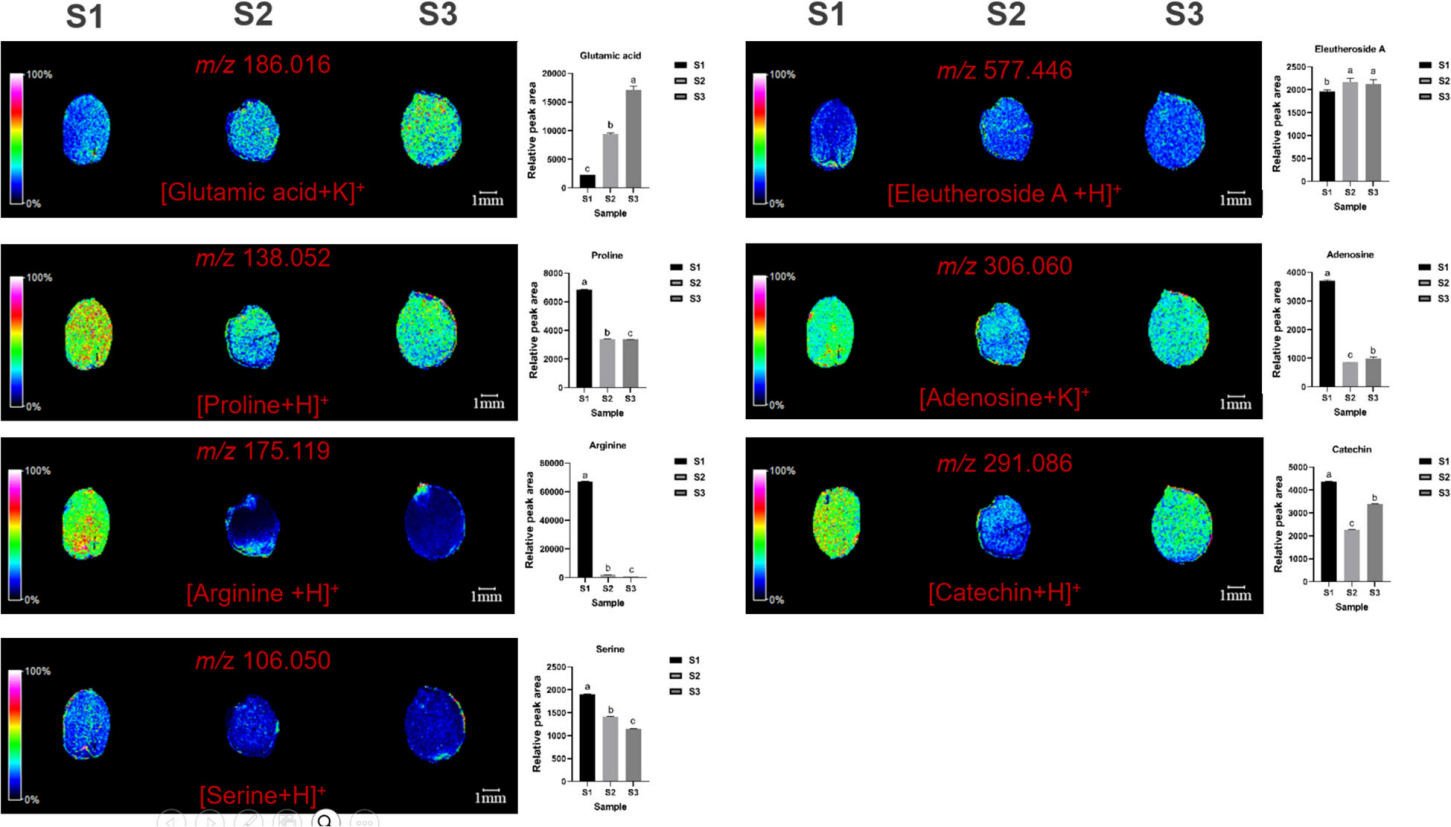
Selected ion maps of seven non-essential amino acids detected from the tissues of ZSS growth in different periods sections by MALDI-TOFTOF MS in the positive-ion mode using 2-MBT as the matrix. A color scale from white to blue indicates a high-to-low sign. S1 was the period of white maturity, S2 was the period of firm ripening and S3 was the period of full ripening.

MALDI imaging also revealed the spatial distribution of seven non-essential amino acids: glutamic acid (*m/z*=186.016, [M+K]^+^), proline (*m/z*=138.052, [M+Na]^+^), arginine (*m/z*=175.119, [M+H]^+^), serine (*m/z*=106.050, [M+H]^+^), eleutheroside A (*m/z*=577.446, [M+H]^+^), adenosine (*m/z*=306.060, [M+K]^+^), and catechin (*m/z*=291.086, [M+H]^+^) (**Figure 6**). Glutamic acid was mainly distributed in the cotyledons, with content increasing over time. Proline was distributed in cotyledons and radicles, with content first decreasing and then increasing. Arginine was the highest throughout the seed and in cotyledons at S1 initially, but gradually decreases, becoming concentrated in the radicle. Serine was mainly distributed in the endosperm, with content first decreasing and then increasing. (S)-coclaurine was mainly distributed in the cotyledons, with content decreasing over time, and becoming very low by S3. Eleutheroside A, adenosine, and catechin were mainly distributed in the cotyledons and endosperm, with content first decreasing and then increasing.

Compared to S1, S3 showed reductions of 45.64% in proline, 98.72% in arginine, 33.23% in serine, and 65.43% in (S)-coclaurine. S2 showed reductions of 45.30% in proline, 96.56% in arginine, 17.96% in serine, and 59.00% in (S)-coclaurine compared to S1. These findings indicate that the content of these compounds generally decreases over time. Conversely, compared to S1, S3 showed increases of 732.92% in glutamic acid. S2 showed increases of 192.94% in phenylalanine compared to S1. This indicates that glutamic acid increase during plant growth. S3 also showed increases of 15.97% in eleutheroside A, 70.43% in adenosine, and 14.80% in catechin compared to S1. S2 showed increases of 18.34% in eleutheroside A, 74.53% in adenosine, and 43.05% in catechin compared to S1 (**Table 1**). Thus, the content of eleutheroside A increases first and then decreases, while adenosine and catechin decrease first and then increase.

### 3.7 Potential quality-associated markers in ZSS at different growth periods discovered by MALDI-MSI combined with OPLS-DA

To harness the capabilities of mass spectrometry imaging for detailed analysis of ZSS quality, ZSS samples representing different growth phases and characterized by quality attributes were examined using MALDI-MSI. Despite commonalities in the spatial distribution of bioactive ingredients across samples, differences in relative signal intensity were observed in smaller tissue regions. The OPLS-DA technique was employed to differentiate ZSS samples from different growth periods and identify key components contributing to these distinctions.

**Figure 7:**
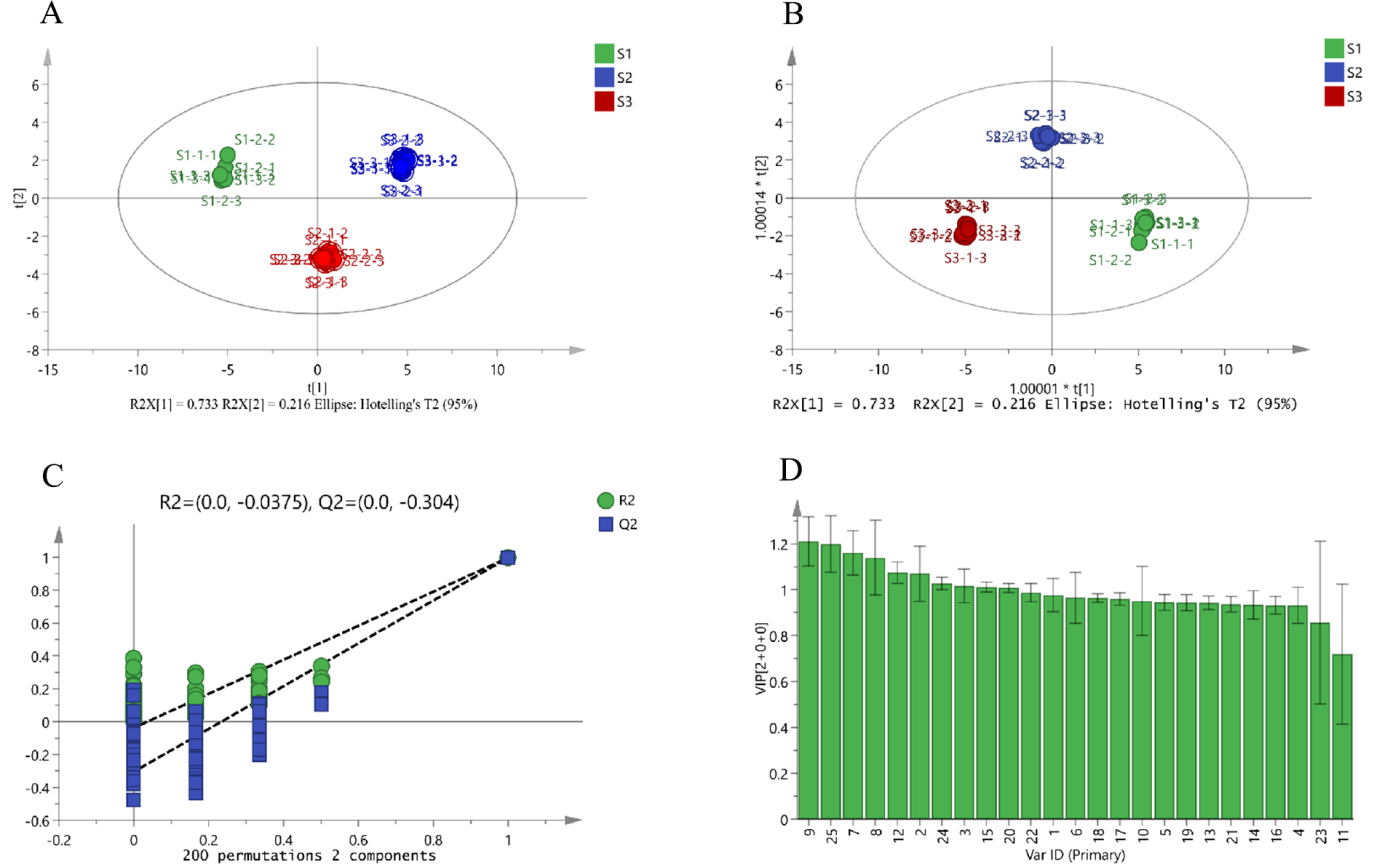
PCA score plot **(A)**, OPLS-DA score plot **(B)**, permutation test plot **(C)**, and VIP value **(D)** of the potential quality-associated markers for ZSS growth in different periods discovered. 1. jujuboside A, 2. jujuboside B, 3. sanjoinenine, 4. spinosin, 5. 6-feruloylspinosin, 6. AGOME, 7. vicenin-2, 8. 6”-p-coumaroylspinosin, 9. 6’’’-sinapoylspinosin, 10. CMBDPM, 11. Dl-Nuciferine, 12. amphibine D, 13. ORTHO-NORNUCIFERINE, 14. glutamic acid, 15. proline, 16. lysine, 17. phenylalanine, 18. methionine, 19. L-isoleucine, 20. arginine, 21. serine, 22. (S)-coclaurine, 23. eleutheroside A, 24. adenosine, 25. Catechin.

The OPLS-DA models, constructed using MSI data from the cross-sectional analysis of the full spectral range (*m/z* 100 to 1,200), elucidated the variations in components during different growth phases. Both Principal Component Analysis (PCA) and OPLS-DA identified disparities in the compounds as ZSS progressed through growth stages (**Figures 7A, B**). The independent variable model parameter (R2X) and dependent variable model parameter (R2Y) were both greater than 0.5, indicating good model stability and predictive ability. The permutation test plot showed that the model did not overfit and had good predictive ability (**Figure 7C**).

The Very Important Projection (VIP) values for 6’’’-sinapoylspinosin, catechin, vicenin-2, 6’’-p-coumaroylspinosin, jujuboside B, amphibine D, adenosine, sanjoinenine, and proline were all greater than 1 (**Figure 7D**). Therefore, these components could be used as potential quality-associated markers to distinguish ZSS growth at different periods.

### 3.8 Relative content of indicative bioactive ingredients

**Figure 8:**
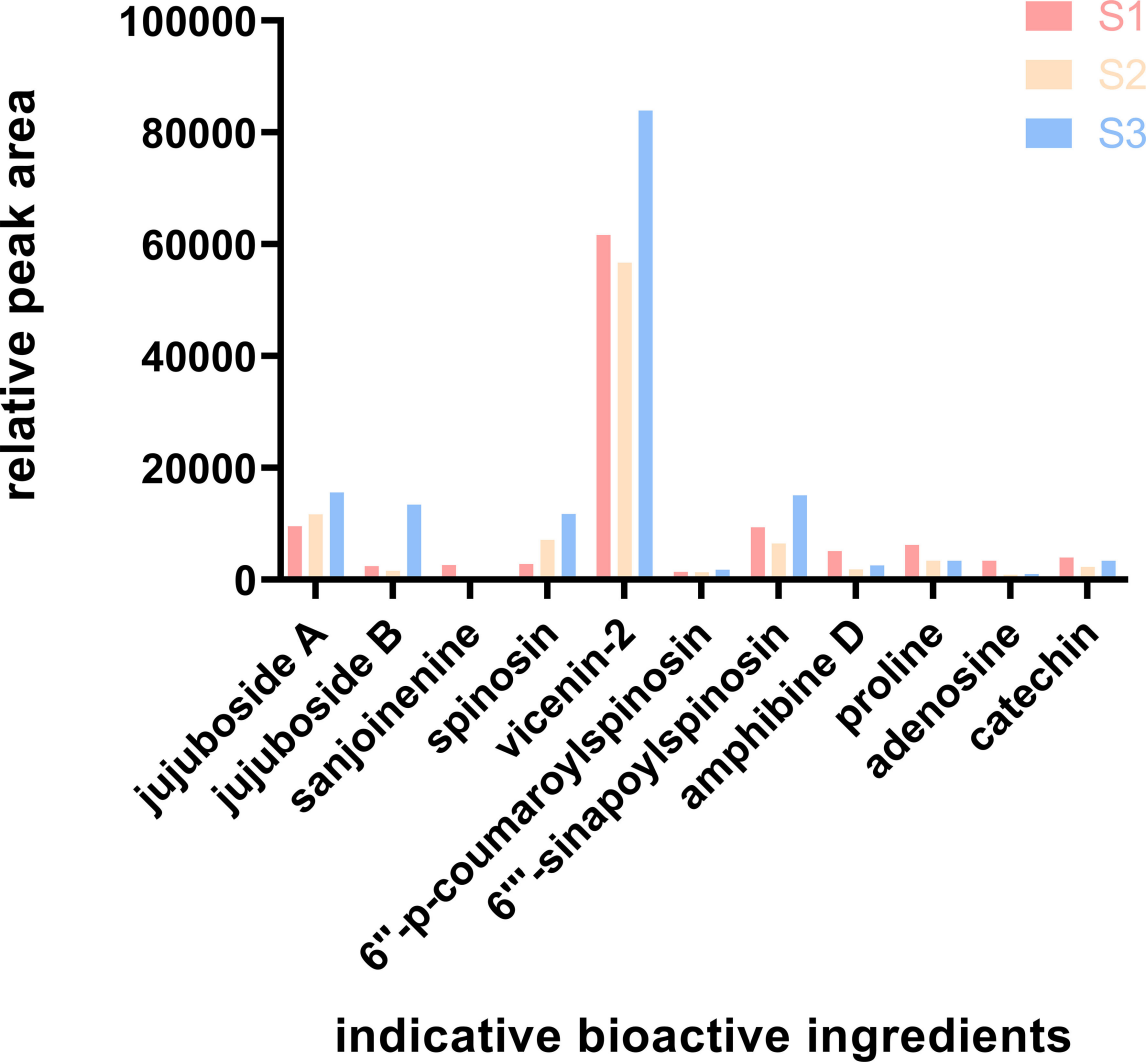
Relative content of the indicative bioactive ingredients. S1 was the period of white maturity, S2 was the period of firm ripening and S3 was the period of full ripening.

This research presents an in-depth analysis of the relative peak areas of nine bioactive constituents with Variable Importance in Projection (VIP) values exceeding unity, scrutinizing their concentration fluctuations throughout the ripening process of ZSS (**Figure 8**). The bar chart data starkly delineate the trend, illustrating an initial decline followed by an ascent in the content of these bioactive components.

In the 2020 edition of the Chinese Pharmacopoeia, jujuboside A and spinosin are stipulated as the quality marker compounds for the acid ZSS. This research, through meticulous peak area analysis, uncovers a consistent increase in the levels of these two constituents throughout the entire growth phase of ZSS.

## 4 Discussion

ZSS is a rich repository of diverse bioactive constituents, including flavonoids, terpenoids, alkaloids, and fatty oils (Li et al., 2020; Jiang et al., 2022b). Their concentrations undergo dynamic changes during the developmental stages of ZSS. A deeper understanding of the maturation process and its impact on the chemical profile of ZSS is pivotal for enhancing its medicinal quality and innovating health products.

Mass spectrometry imaging (MSI) provides an intuitive and vivid depiction of traditional Chinese medicine, offering superior spatial information on natural components and high-throughput analysis potential (Chinese Pharmacopoeia Commission, 2020; Zhao et al., 2017). This research leveraged MALDI-MSI to map the spatial distributions and analyze the distribution changes of biologically active substances during ZSS development. The MALDI-MSI results elucidated the spatial dynamics of terpenoids, flavonoids, alkaloids, and amino acids at various growth stages of ZSS.

Previous researchs have indicated a decrease in the content of terpenoids and flavonoids during ZSS development, reaching the lowest point at fruit ripening, aligning with our findings on flavonoid content during the liquid diversion phase. Jiang et al. posited that the content of flavones and saponins increases with fruit maturity, influenced by the biosynthesis of secondary metabolism (Li et al., 2022b). The content of jujuboside A and spinosin, primarily distributed in the radicle and endosperm, gradually increases over time, underscoring their importance as bioactive markers of ZSS quality, as per the 2020 Chinese Pharmacopoeia (Guo et al., 2023).

The levels of dl-Nuciferine, amphibine D, ortho-nornuciferine, and (S)-coclaurine, key alkaloids in ZSS, exhibit distinct trends, with dl-Nuciferine showing a gradual increase, amphibine D a fluctuating pattern, and both of ortho-nornuciferine and (S)-coclaurine a steady decline. They were mainly distributed in the endosperm and cotyledon. Dl-Nuciferine plays an important role in the treatment of inflammation-related diseases. Xue et al. also obtained the result of the decreased growth content of dl-Nuciferine with the growth of ZSS. Coclaurine exerts its influence on the serotonergic and GABAergic synaptic pathways which may contribute to the therapeutic effects of ZSS in insomnia (Bian et al., 2021). It is a precursor to the synthesis of various alkaloids in the biosynthetic pathway. In this research, the content of (S)-coclaurine gradually decreased, suggesting that it may be used to synthesize other alkaloids in ZSS during the growth of ZSS.

The overall levels of glutamic acid, lysine, phenylalanine, and eleutheroside A were elevated in ZSS, while L-isoleucine, methionine, proline, arginine, serine, adenosine, and catechin showed a general reduction. The increase in drug-effective amino acids like lysine, phenylalanine, and glutamic acid indicates their contribution to the medicinal value of ZSS. Amino acids significantly shape the flavor of food, although they do not themselves directly give it a specific taste (Tang et al., 2021; Moran et al., 2015). The alteration in the composition and content of free amino acids can impact the nutritional value and flavor of fruits and affect the absorption and utilization efficiency of food nutrition (Yu et al., 2024). Based on their chemical properties, amino acids can be classified as types with sweet, umami, bitter, and aromatic properties. These different amino acid classes work together to give foods their unique flavor and highlight their edible properties (Hong et al., 2024). Glutamic acid and lysine, flavor amino acids (Liu et al., 2023b), elevated the levels that can intensify the umami taste in ZSS. A boost in phenylalanine content enhances their aromatic fragrance (Zhao et al., 2024). Decreasing the quantities of arginine, methionine, and isoleucine diminishes the bitterness, which in turn enhances the flavor profile of the ZSS. These adjustments are helpful to enhance the edible of ZSS.

To investigate further changes in ZSS content, they were distinguished using PCA and OPLS-DA for different growth periods (Alharbi et al., 2021; Lippold et al., 2021). The PCA and OPLS-DA score plot offers a clear visual representation of how various ZSS samples are arrayed within the principal component space. These samples across different periods have coalesced into distinct clusters on the score plot, signifying the robust consistency of the sample data. The OPLS-DA With both the R² and Q² values surpassing the 0.5 benchmark, the model demonstrates not only a robust fit to the data but also an impressive predictive capability.

VIP values are a very useful tool in the OPLS-DA model to help researchers identify and interpret patterns and trends in the data (Wang et al., 2023c). In this research, nine variables exhibited VIP values exceeding the threshold of 1, indicating their significant influence on the model. They are identified as potential markers indicative of the quality of ZSS. These nine bioactive ingredients displayed a fluctuating pattern, initially decreasing and then increasing during the growth of ZSS.

In this study, the MALDI technique was employed to characterize the chemical constituents in ZSS, successfully identifying 25 compounds. However, due to limitations in experimental conditions, the analysis of certain compounds did not achieve optimal results. Specifically, the instrument’s performance and the lack of comprehensive chemical reference standards constrained our detection primarily to higher fragment ions (m/z), while several low fragment ion (m/z) compounds could not be effectively detected. Additionally, seed tissue sections of ZSS were used as samples, which are rich in high concentrations of K^+^ and Na^+^, facilitating their binding to multiple compounds during MSI. However, when these compounds are present independently in the external environment of the seed, they exhibit weaker binding affinity to K^+^ and Na^+^ and lack appropriate standards for comparison. Future work will focus on optimizing the experimental protocol and enhancing experimental conditions to acquire more comprehensive and robust data, thereby providing stronger support for our findings.

## 5 Conclusions

This research successfully applied a MALDI-MSI method for the direct analysis of bioactive ingredients in ZSS. The spatial distribution of terpenoids, flavonoids, alkaloids, and amino acids in ZSS tissues was mapped, and their changes during different developmental stages were investigated. Nine bioactive ingredients, identified through multivariate statistical analysis, serve as potential quality indicators for ZSS at various growth periods, establishing a scientific foundation for the quality evaluation of ZSS.

## Data Availability

The original contributions presented in the study are included in the article/**Supplementary Material**. Further inquiries can be directed to the corresponding authors.
