# Supplementary material for: Matrix-assisted laser desorption/ionization mass spectrometry imaging analysis revealed the spatial distribution of metabolites during Ziziphi Spinosae Semen at different growth periods
Source: Front Plant Sci. 2025 Feb 21;16:1510310. doi: 10.3389/fpls.2025.1510310 (PMC11885241; doi:10.3389/fpls.2025.1510310)
Supplement: Supplementary file 1 [file DataSheet1.docx]

Supplementary Material

**
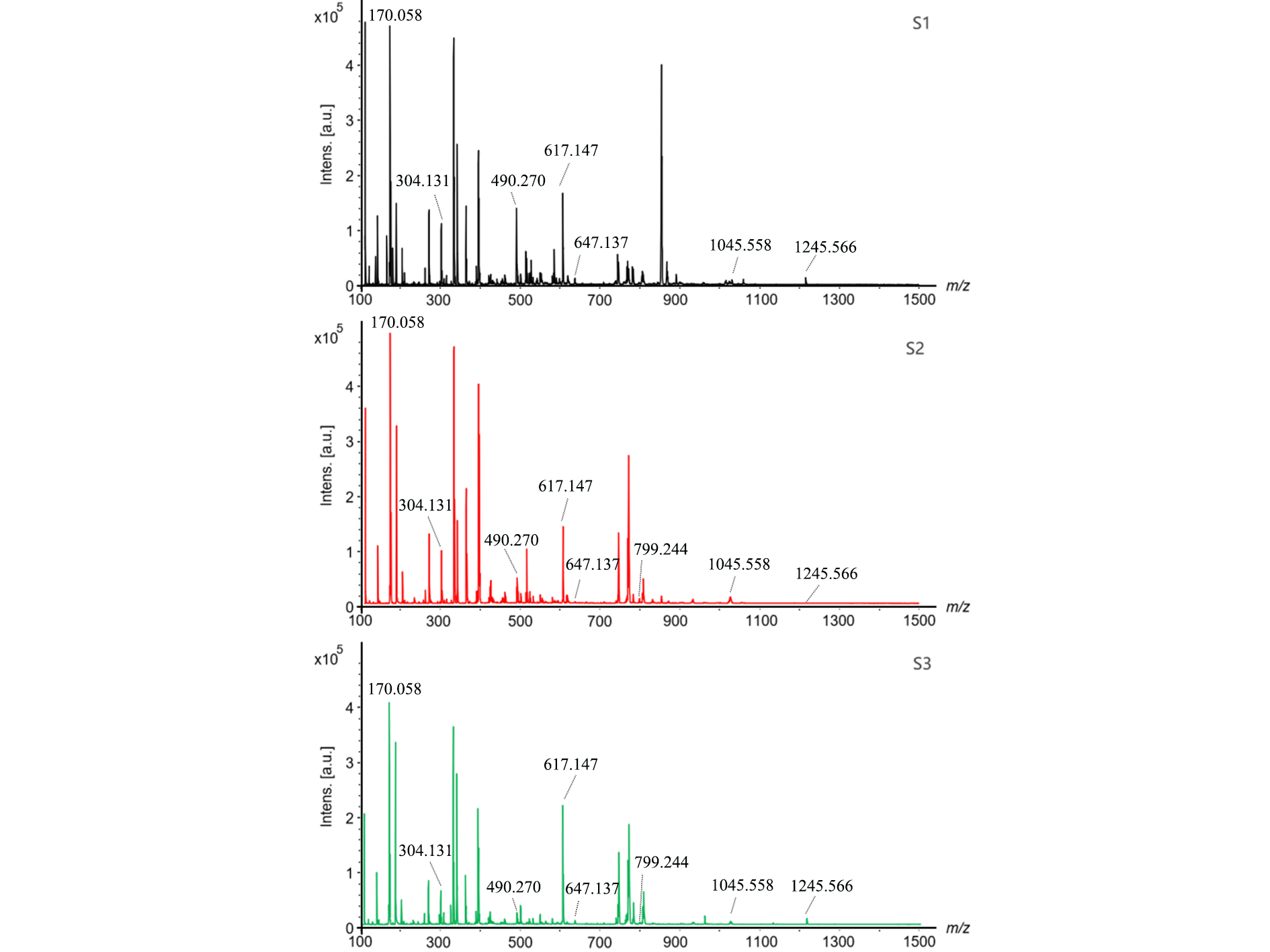
**

**Figure S1.** Comparison of mass spectra acquired from laser irradiation of tissue sections of ZSS growth in different periods by (+) MALDI-TOFTOF MS using 2-MBT as the matrix. S1 was the White maturity stage, S2 was the firm ripening stage and S3 was the full ripening stage.


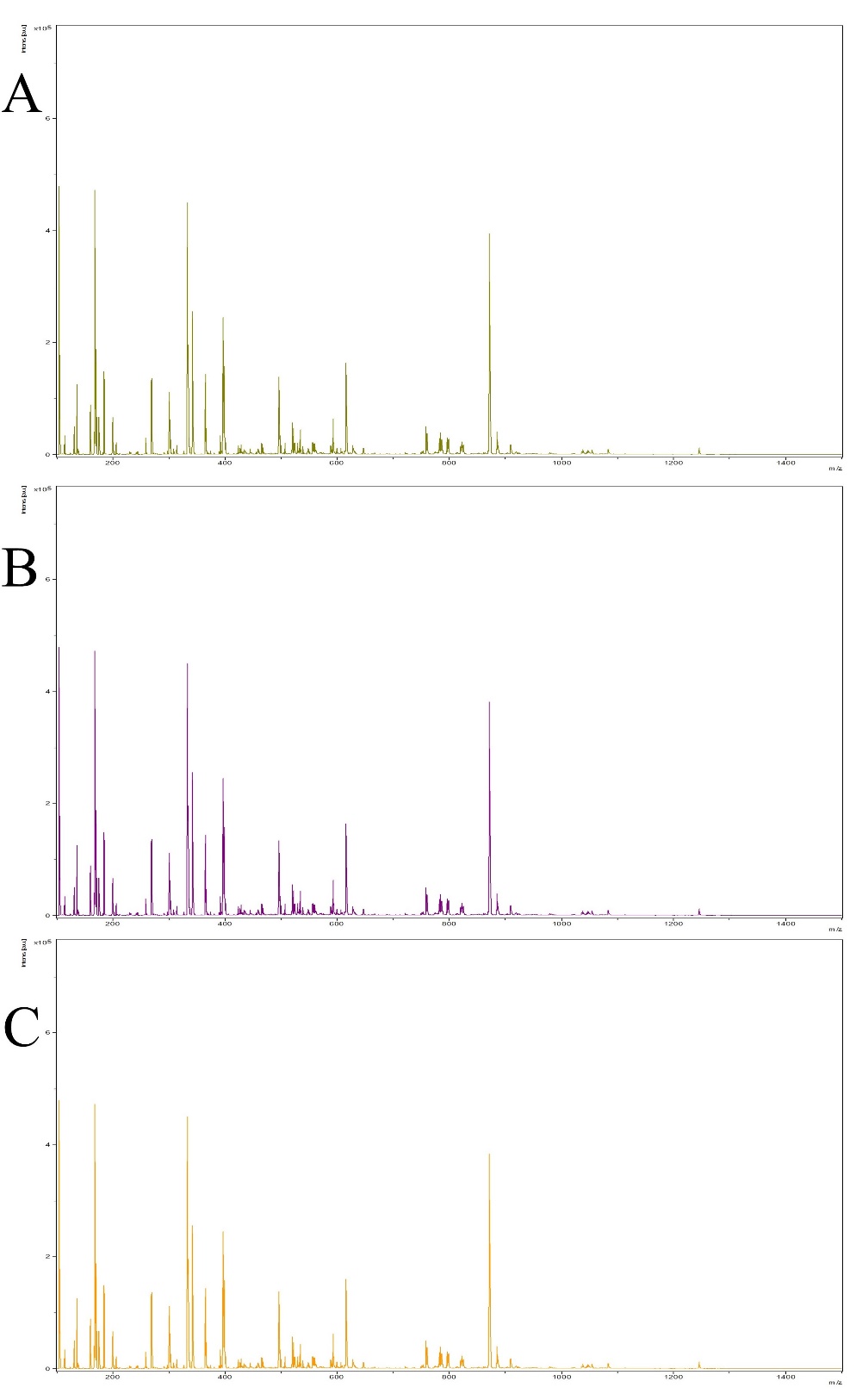


**Figure S2.** Interday reproducibility validation. A, 0 day; B, 2day; C, 3day*.*
